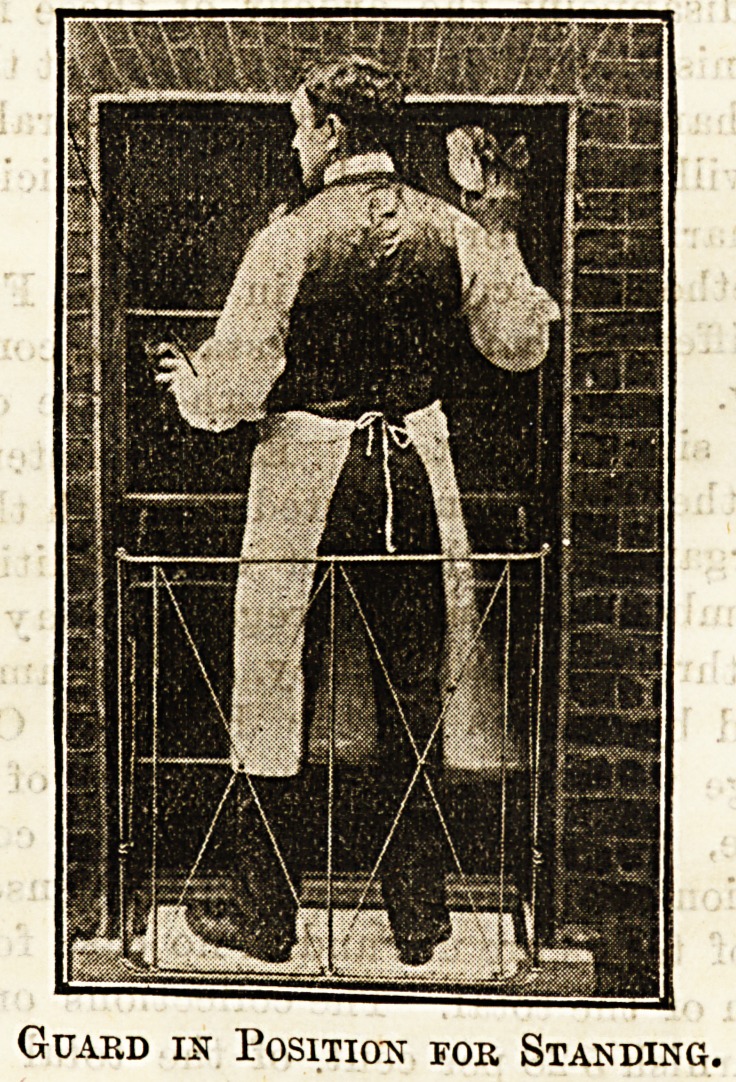# Practical Departments

**Published:** 1894-03-24

**Authors:** 


### PRACTICAL DEPARTMENTS.

### THE VICTORIA SAFETY WINDOW CLEANING GUARD.

New safety windows and appliances for facilitating window cleaning are everywhere coming to the fore, so much public attention having been lately drawn to the question of how to minimise the undoubted dangers and risks attendant upon the outside cleaning of windows.

In cases where a wholesale alteration in shape and construction is not possible or desirable some sort of safety guard should certainly be used, and we give below illustrations of a very strong and convenient contrivance, a sort of portable balcony, which is being brought out by the Victoria Safety Appliance Company, of 17, Victoria Street, S.W.

The guards may be had in two sizes, one for use where standing is necessitated, the other where it is possible to pull the top sash down for cleaning purposes, the first being somewhat higher and stronger. They are absolutely simple in

Guard in Position for Sitting.

make, and exceedingly strong, while light enough to be easily moved from one window to another. The guard is made of wrought steel and iron wire, its weight being not more than 15lb. to 20 lb., and the price is sufficiently moderate, ranging from 13s. to £1 3s.

The adjustment is quite easy. The guards must, of course, be made to fit the window, and are firmly secured on the inside by an iron bar run through the top of the two inside uprights and projecting side arms, both these being covered

Guard in Position for Standing.

with indiarubber to avoid any injury to paint, &c. Where any difficulty is experienced owing to the stone window sill being too low for the bottom of the guard to rest upon, a board of the necessary thickness should be placed on the sill before fixing the guard.

There is no doubt that such an arrangement as this makes accidents of the kind so much commented on of late practically impossible, unless the window-cleaner had a particular leaning towards suicide. It would be difficult to feel even a little giddy with so firm a support on all sides.

Some experiments were made recently at the Victoria Mansions to test the usefulness of the window guards, and its practical advantages were amply demonstrated. Really no house should be without so simple and inexpensive a means of preventing most horrible accidents.

Mr. Anidjah, who has invented and patented this guard, is also the inventor of an excellent fire escape.

## Figures and Tables

**Figure f1:**
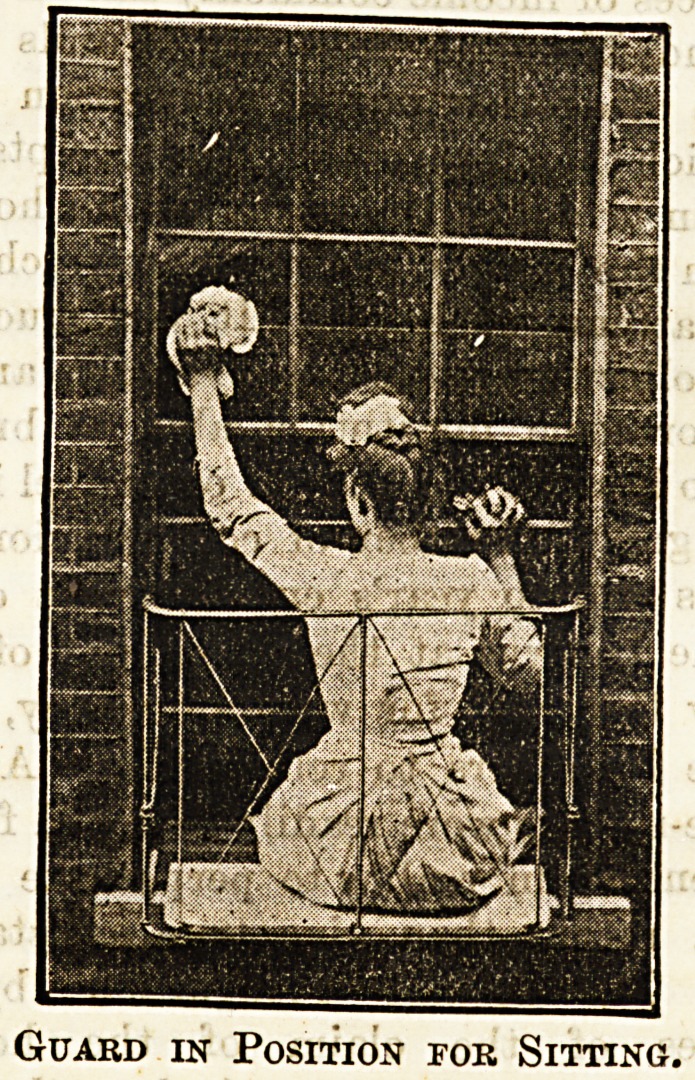


**Figure f2:**